# Wellens’ Syndrome Presenting as Epigastric Pain and Syncope: An Unusual Presentation

**DOI:** 10.7759/cureus.6877

**Published:** 2020-02-04

**Authors:** Temitope Ajibawo, Alexander Andreev, Sonu Sahni

**Affiliations:** 1 Internal Medicine, Brookdale University Hospital Medical Center, New York, USA

**Keywords:** electrocardiography, cardiac catheterization, myocardial infarction, wellens' syndrome, left anterior descending stenosis

## Abstract

Wellens’ syndrome, also regarded as left anterior descending coronary T-wave syndrome, is an electrocardiography (EKG) pattern that indicates critical proximal left anterior descending artery (LAD) stenosis. It is characterized by deeply inverted T-waves or biphasic T-waves in the anterior precordial chest leads in a patient with unstable angina. Patients typically present with symptoms consistent with acute coronary syndrome. We present a unique case of Wellens’ syndrome with no angiographic findings of significant stenosis in the proximal LAD but with significant occlusion of the proximal circumflex artery and initial presentation with a chief complaint of epigastric pain and syncope. Physicians need to recognize these characteristic EKG changes during the pre-infarction stage, as they represent myocardial necrosis. Many of these patients eventually develop extensive anterior myocardial infarction with marked left ventricular dysfunction and death if coronary angiography and coronary revascularization are not performed within a few weeks. If Wellens’ is seen, patients should undergo urgent cardiac catheterization.

## Introduction

Wellens’ syndrome, also regarded as left anterior descending coronary T-wave syndrome, was first described by de Zwaan et al. in the late 20th century, who recognized specific precordial T-wave changes in the setting of anticipant changes and, subsequently, anterior wall myocardial infarction (MI) in a subset of patients with unstable angina [1]. Wellens’ syndrome criteria include deeply inverted T-waves or biphasic T-waves in the anterior precordial chest leads, i.e. V2 and V3 (may involve V1-V6), in a patient with unstable angina [2]. These T-wave changes on electrocardiogram (EKG) may persist for hours to days and are linked to a possible underlying obstruction in the proximal left anterior descending (LAD) coronary artery [2]. The typical presentation is that of unstable angina, which includes chest pain, which may be present at rest, chest tightness, and diaphoresis, along with other commonly seen symptoms of acute coronary syndrome. Wellens’ syndrome is imperative to recognize and to act on expeditiously as it often represents evolving myocardial necrosis and may be missed, as there are often insignificant elevations in cardiac enzymes if any. Herein, we present a case of Wellens’ syndrome with a clinically confusing presentation of abdominal pain, syncope with precordial T-wave inversions, along with a review of the literature.

## Case presentation

**Figure 1:**
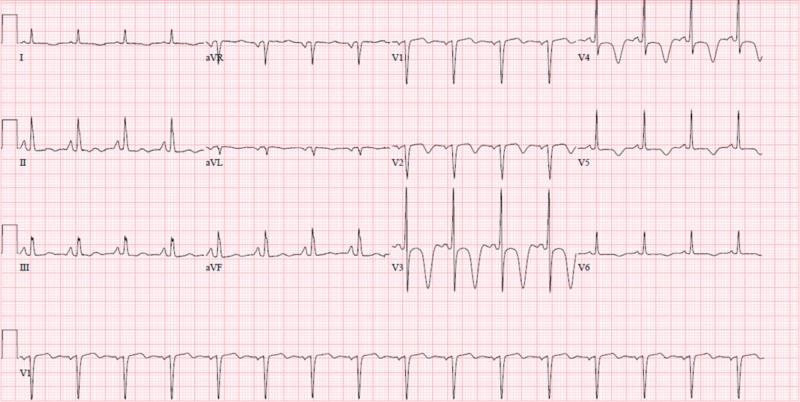
EKG showing T-wave inversions in V2-V6 with deep symmetric inverted T-waves in leads V3 and V4 EKG: electrocardiogram

A 58-year-old African-American male, an active smoker, presented to the Brookdale University Hospital Medical Center emergency room with a chief complaint of an episode of syncope 45 minutes before presentation. He also reported a week-long history of generalized fatigue and epigastric pain associated with nausea and multiple episodes of non-bloody, non-bilious vomiting. Upon further questioning, the patient reported that while walking, he felt dizzy and passed out for about a minute as witnessed by a bystander. The patient denied any chest pain, diaphoresis, dyspnea, left arm pain, or lower extremity edema. Past medical history included diabetes mellitus, hypercholesteremia, cigarette smoking (four to five cigarettes/day x 40 years), marijuana use (last used five days prior to presentation), chronic obstructive pulmonary disease, and cocaine use (last used more than a week ago). He also reported drinking two to three beers per week. Initial vital signs on presentation showed a blood pressure of 158/82 mmHg, respiratory rate of 18 breaths per minute, heart rate of 110 beats per minute, and oxygen saturation on room air of 97%. On examination, the patient was fully oriented, the abdominal examination revealed a soft, slightly tender epigastric region. Other examination findings were unremarkable. For reported syncope, the patient underwent computed tomography of the head, which was negative for any acute intracranial pathology. Initial electrocardiogram (EKG) obtained on admission showed normal sinus rhythm, deep symmetrical T-wave inversions in leads V3 to V4, and prolonged QTc representative of Wellens’ syndrome as shown in Figure 1.

Initial laboratory values are shown in Table 1. The initial troponin level was noted to be 0.016 ng/ml, which is negative for acute coronary syndrome.

**Table 1:** Initial laboratory values on admission AST: aspartate aminotransferase; ALT: alanine aminotransferase

Laboratory Test (Normal Range)	Initial Values
Hemoglobin (11.4-15.5 g/dL)	18.5 g/dL
Hematocrit (37.0-43.7%)	55.4%
White blood cell count (4.5-10.2 x 10^9^/L)	8.5 x 10^9^/L
Platelet count(180-401 x 10^9^/L)	270 x 10^9^/L
Troponin (0.00- 0.034 ng/mL)	0.016 ng/mL
Blood urea nitrogen (7.0-17.0 mg/dL)	34 mg/dL
Creatinine (0.52-1.04 mg/dL)	1.30 mg/dL
Sodium (133-145 mEq/L)	127 mEq/L
Potassium (3.5-5.1 mEq/L)	4.3 mEq/L
AST/ALT (14-36 U/L / 9-52 U/L)	164/185 U/L
Lactate (0.70-2.10 mmol/L)	2.40 mmol/L
Creatine kinase (55-170 U/L)	36 U/L

**Figure 2:**
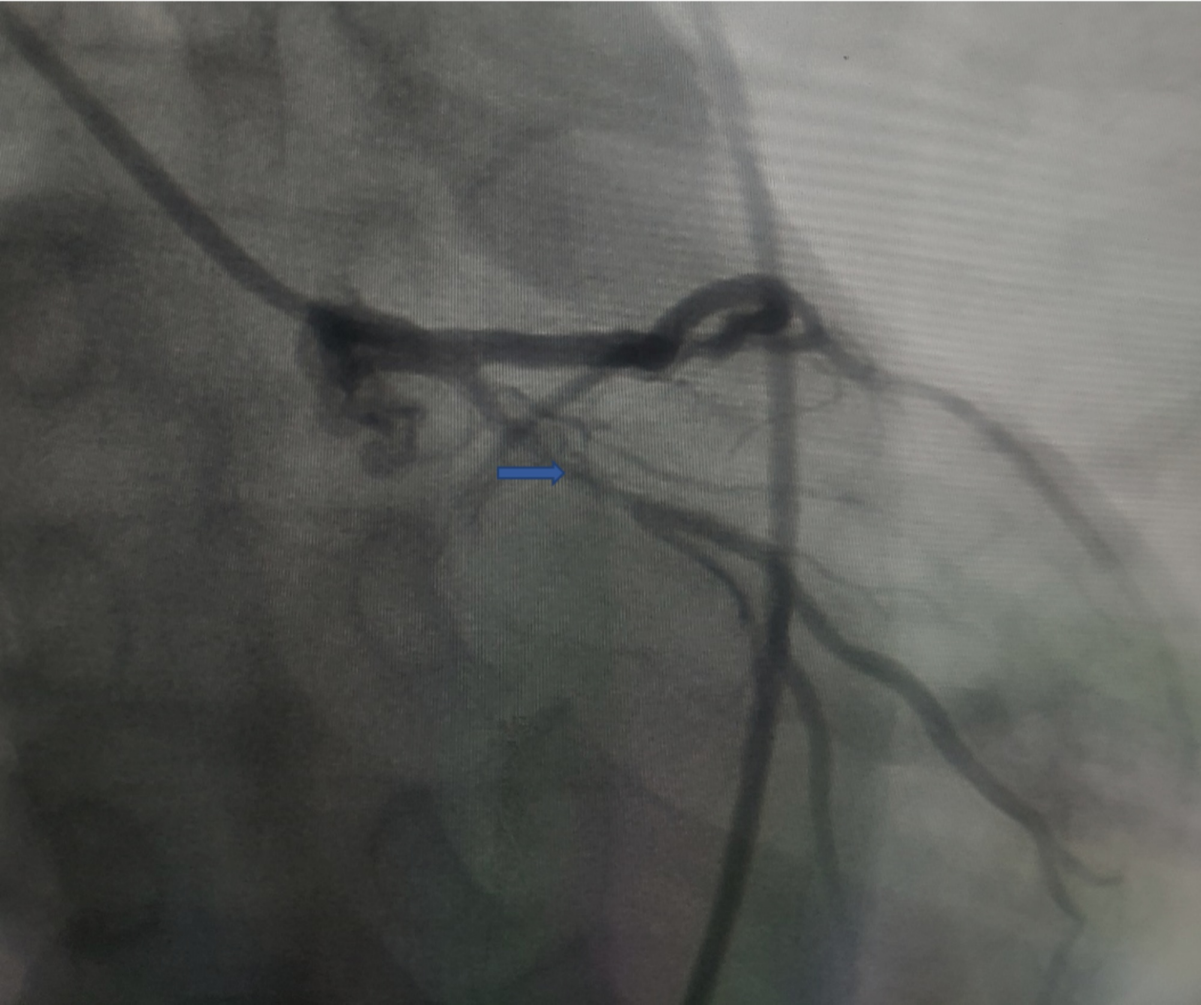
Coronary angiogram showing 85% stenosis of the proximal circumflex

Echocardiography did not show any diagnostic regional wall abnormality; however, it did show mild left ventricle wall thickness, concentric hypertrophy, grade 1 diastolic dysfunction, and a left ventricular ejection fraction of 50%. Due to deep symmetric T-waves and symptoms disconcerting for an underlying coronary artery lesion, the patient was scheduled for urgent cardiac catheterization. The patient was loaded with 325 mg of aspirin as well as 180 mg of ticagrelor and was started on therapeutic enoxaparin. He was admitted to the coronary care unit (CCU). The patient underwent left heart catheterization, which showed 85% stenosis of the proximal circumflex artery, as shown in Figure 2, and mild atherosclerosis disease in the LAD.

**Figure 3:**
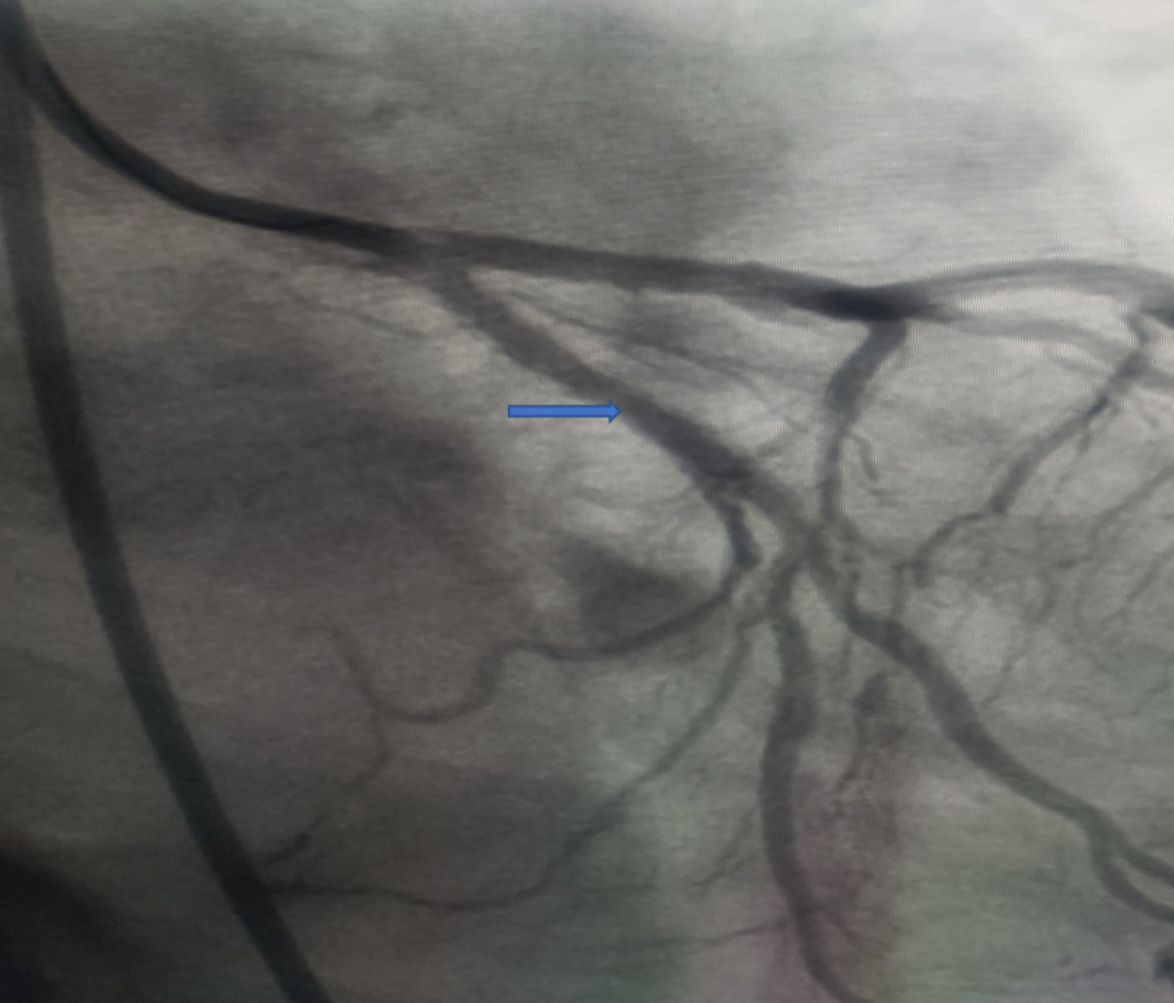
Coronary angiogram showing excellent angiographic appearance with 0% residual stenosis in the proximal circumflex following the placement of a drug-eluting stent

The 85% proximal circumflex lesion was treated with balloon angioplasty and the placement of a drug-eluting stent (DES). Following the intervention, there was 0% residual stenosis in the proximal circumflex artery, which is shown in Figure 3.

**Figure 4:**
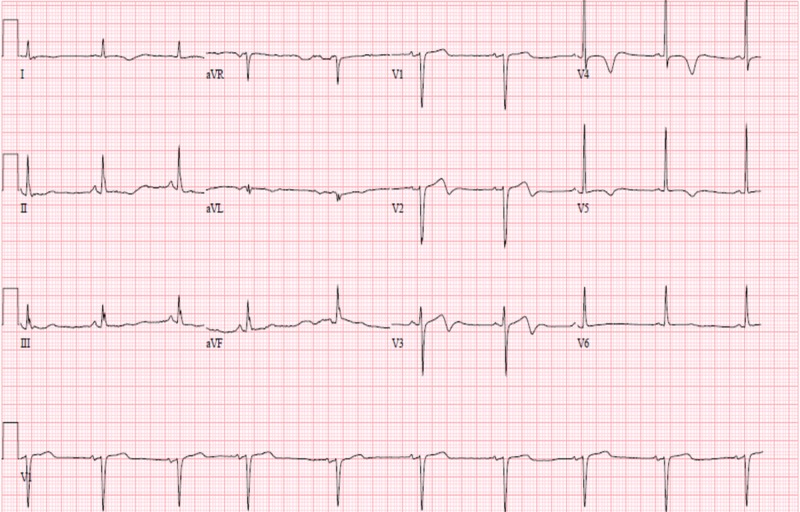
Shows biphasic T-waves in V2 to V3 and T-wave inversion in V4 and V5

EKG on discharge, two days after stent placement is shown in Figure 4, and it demonstrates biphasic T-waves in leads V2, V3, and T-wave inversions in V4 and V5.

He was discharged home on a high-intensity statin, beta-blocker, and dual antiplatelet therapy (aspirin and ticagrelor).

## Discussion

Wellens’ syndrome was first described in 1982, de Zwann and colleagues recognized that 26 of 145 (18%) patients admitted for unstable angina demonstrated characteristic EKG patterns [1]. Furthermore, it was observed that 75% of these 26 patients that did not undergo urgent coronary angiography developed extensive anterior wall MI within a few weeks of admission [1]. In a larger study by de Zwaan et al., it was observed that all 180 out of the 1260 patients admitted for unstable angina with these characteristic EKG changes had stenosis of the LAD, ranging from 50% to complete obstruction [3]. Criteria for the diagnosis of Wellens’ syndrome include the following: absent precordial Q waves, normal precordial R-wave progression, deeply inverted or biphasic T-waves in V2 and V3, insignificant ST-elevation usually (<1 mm) and have normal or minimally elevated cardiac serum enzymes [2]. These EKG features are apparent when patients are asymptomatic or present with non-cardiac related complaints. Wellens’ T-wave abnormality has two different patterns, which are (1) the type A, less common, biphasic T waves and (2) the type B, the more common deep and symmetrically inverted T-wave pattern [1,3]. Our patient presented atypically with epigastric pain and syncope; though not isolated, this is uncommon.

In a report by Mufti et al., an 87-year-old female who presented for an elective skin graft procedure was admitted to the telemetry floor due to atrial fibrillation on admission EKG. Telemetry showed EKG concerning for Wellens’ syndrome, and she underwent catheterization, which showed 60%-70% occlusion in the mid LAD with no significant stenosis in the classical proximal LAD [4]. More recently, Ghizzoni reported a case of a 46-year-old female who presented with symmetric and deep T-wave inversion in V2-V6 (type B Wellens’ syndrome) but left heart catheterization showed 95% stenosis of the mid LAD [5]. Table 2 lists some recent case reports of the atypical presentations of Wellens' syndrome, including the case reports discussed above.

**Table 2:** Recent case reports of atypical presentations of Wellens’ syndrome LAD: left anterior descending artery

Author	Clinical presentation	Angiographic findings
Yasin et al. 2016 [6]	Presented with syncope, and Wellens’ deep T-wave inversion in V1 to V3 but no chest pain.	100% occlusion of the proximal LAD
Kyaw et al. 2018 [7]	Presented with isolated throat pain, Wellens’ deep symmetric T-wave inversion in right precordial leads on telemetry. No chest pain	90% occlusion of the proximal LAD
Mufti et al. 2018 [4]	Presented for an elective skin graft. Found to have Wellens’ T-wave inversions in V2 to V6, I and aVL. No chest pain.	60-70% occlusion in the mid LAD
Ghizzoni et al. 2019 [5]	Presented with intermittent chest pain and Wellens’ T-wave inversion in precordial leads.	95% occlusion of the mid LAD
Shaukat et al. 2019 [8]	Presented with left jaw pain and biphasic T-waves in V1 to V3 suggestive of Wellens’ syndrome. No chest pain	40% occlusion of the proximal-mid LAD

Furthermore, it is important to discuss pseudo-Wellens’ syndrome, a clinical phenomenon in which EKG patterns are suggestive of Wellens’ syndrome but angiogram shows normal coronaries. Pseudo-Wellens’ syndrome has been reported in heavy cannabis, cocaine, and phencyclidine use [9-13]. Pseudo-Wellens’ syndrome has also been implicated in the clinical settings of acute cholecystitis, myocardial bridging, Takotsubo cardiomyopathy, pulmonary embolism, and vasospastic angina [14-17]. A biphasic T-wave suggestive of Wellens’ syndrome has also been reported to be associated with left ventricular hypertrophy [18].

Our case illustrates Wellens’ syndrome variant presenting atypically. The coronary artery lesion was present in the proximal circumflex artery instead of the typical proximal LAD. This patient presented with syncope and non-specific symptoms of nausea, vomiting, fatigue, dizziness, epigastric pain, and syncope without the typically reported chest pain. It is also important to note that cardiac stress tests are contraindicated in these patients, as it delays the diagnosis of severe proximal LAD disease, and it can induce myocardial infarction in an already critically stenosed artery. This case also represents an atypical presentation of ACS, as it unusual for it to present with syncope and epigastric pain. All the sequential troponin levels drawn at least six hours apart before the cardiac catheterization remained negative.

## Conclusions

Although this case presentation represents a variation of Wellens’ syndrome, it is crucial that health care providers recognize this ominous EKG pattern, as it is often missed or misread. It is also imperative that health care providers be aware of atypical presentations of Wellens’ as demonstrated in our case. Once suspected, urgent cardiac catheterization is recommended over stress testing, as underlying coronary artery disease may be present.
